# Good acceptability of HIV, HBV, and HCV screening during immigration medical check-up amongst migrants in France in the STRADA study

**DOI:** 10.1371/journal.pone.0235260

**Published:** 2020-06-26

**Authors:** Martin Duracinsky, Frédérique Thonon, Sonia Bun, Imène Ben Nasr, Aïchata Fofana Dara, Sabrina Lakhdari, Laurence Coblentz-Baumann, France Lert, Svetlane Dimi, Olivier Chassany

**Affiliations:** 1 Université Paris-Diderot & Inserm, UMR1123, Patient-Centered Outcomes Research, Paris, France; 2 Hôpital Bicêtre, Service d’infectiologie, Le Kremlin Bicêtre, France; 3 AP-HP, hôpital Hôtel-Dieu, Unité de Recherche Clinique en Economie de la Santé (URC-ECO), Paris, France; 4 Agence nationale de recherche sur le sida et les hépatites virales (ANRS), Paris, France; 5 Hôpital Foch, Service d’Hépatologie, Suresnes, France; University of London, UNITED KINGDOM

## Abstract

**Introduction:**

The prevalence of HIV, hepatitis B, and hepatitis C amongst migrants in France is high. Thus, effective screening and follow-up is needed. The mandatory medical check-up for residency application is an opportunity to offer rapid HIV and hepatitis testing. The main objective of the STRADA study is to create a feasible and acceptable screening strategy for migrants. Within the STRADA study, this qualitative research examined the acceptability of conducting screening tests in the context of residency application.

**Methods:**

We conducted a qualitative study amongst legal migrants over 18 years of age with sufficient knowledge of the French, English, or Arabic language. Interviews were performed following a semi-structured interview guide of open-ended questions. Interviews were transcribed verbatim and subsequently analyzed through thematic analysis.

**Results:**

We interviewed 34 migrants. Mean age was 32.6 (min-max: 19, 59) years. The participants’ region of origin was mostly Sub-Saharan Africa and the main reason for migrating to France was family reunification. Migrants' acceptability of HIV and hepatitis testing was high. Participants who accepted testing indicated a benefit for individual health and to avoid transmission. Most preferred rapid tests; reluctance was related to anxiety about the immediate results and the perceived reliability of rapid tests. Migrants' knowledge about HIV was satisfactory, but inadequate for hepatitis. Screening in the context of a compulsory medical visit did not present an obstacle for acceptability. Some expressed concern in the case of HIV but when explained, the independence between obtaining the residence permit along with screening and access to medical care was well understood.

**Discussion:**

Medical check-ups at immigration centers is an opportunity to screen for HIV and hepatitis which is considered acceptable by migrants. Informing migrants that test results do not affect residency applications, and incorporating their preferences, are all important to optimize the acceptability of screening.

## Introduction

Infectious diseases such as HIV, hepatitis B (HBV), and hepatitis C (HCV) are an unresolved issue amongst migrants in France. Indeed, in 2014, amongst the 6600 new cases of HIV discovered, half of them occurred in foreigners living in France. Hepatitis B and C also have a high prevalence amongst migrants. A survey estimated an HCV prevalence of up to 10.2% and a HBV prevalence of up to 43.6% amongst migrants from high endemicity countries [1].

In addition to a high prevalence of HIV, HBV, and HCV, migrants also face the issue of late diagnosis, despite access to free testing and treatment, regardless of income or migration status. Some reasons for late diagnosis have been suggested, such as cultural taboo and lack of knowledge about HIV [2,3].

Late diagnosis of HIV, HBV, and HCV is particularly problematic. Early diagnosis has obvious benefits, both for the individual (e.g., earlier access to care with a better life expectancy) and for the community (e.g., prevention of outbreaks). In regard to HIV, treated patients with an undetectable viral load do not transmit the virus [4,5]. This is in line with the UNAIDS 90-90-90 target, meaning for the first step of the cascade, 90% of people living with HIV should be aware of their status [6].

In France, all legal migrants undergo a mandatory health check-up at the migration office (*Office Français de l’Immigration et de l’Intégration*—OFII) upon arrival in France or when they obtain a residency permit [7]. This free-of-charge check-up includes a chest X-ray screening for active pulmonary tuberculosis, a review of vaccinations, a vision test, a measure of body mass index, and a glycemic test. Currently, no HIV or viral hepatitis screening is offered to migrants as part of this medical check-up. Guidelines recommend that every person should be tested for HIV, hepatitis B, and hepatitis C at least once during their lifetime, regardless of risks, and that migrants are tested for HIV at least once a year [8]. Combining the three tests together is also recommended [9].

Offering screening of these three viral diseases as part of the medical check-up is an opportunity to test a wide range of migrants who otherwise wouldn't be screened. For those who test seropositive, this check-up provides earlier access to effective treatment free of charge.

This screening is not mandatory thus individuals should be given the option to decline or accept such testing. In order to maximize screening rates, questions of the acceptability of screening during the medical examination should be addressed. Thus, it is import to convey to migrants undergoing the visit that they should not feel obliged but rather motivated to be screened. It is also important that migrants understand that the result of these tests remain confidential. They should know that in the event of a positive result, they will be able to access free treatment in France, and that a positive result is no ground for refusing a residence permit. Finally, better understanding the migrant’s preferences of the conditions for performing this screening is important for ensuring its feasibility and acceptability.

The STRADA study evaluates the validity of an electronic risk-factor self-report questionnaire (named “TROD screen”) for a targeted screening strategy of HIV, HBV, and HCV (with rapid screening tests) among migrants. This paper reports the results of the first part of the STRADA study, i.e. the analysis of the acceptability of introducing rapid testing and of a risk-factor screening questionnaire among migrants during mandatory medical check-up at an OFII migration office. The questions focused on migrants’ knowledge of these infectious diseases, their perception on HIV and viral hepatitis testing and on rapid testing, and their opinion on offering testing in the premises of OFII migration offices during the medical check-up.

## Methods

We conducted a qualitative study (between October 2016 and March 2017) consisting of a series of interviews with legal migrants undergoing the mandatory medical check-up at local OFII migration offices.

### Participants

Migrants undergoing the mandatory medical check-up were selected at five local migration offices in three different regions of France. The recruitment was carried out differently according the organization of the office. In two offices, the physician or the nurse invited every migrant during medical consultation to participate in the study. In three offices, the study was presented to all migrants present at the beginning of the day and volunteers were invited to come forward.

Participants included were over 18 years of age and had a sufficient knowledge of the French, English, or Arabic language. We aimed to achieve diversity in terms of sex, age, country of origin, and education level. The sample size was chosen to ensure data saturation, i.e. when new interviews did not add any new relevant information.

### Data collection

Interviews were performed following a semi-structured interview guide of open-ended questions based on a literature review exploring the following themes: knowledge about HIV and hepatitis B and C and its treatments, attitude towards HIV and hepatitis testing, knowledge and opinion on rapid testing, and attitude towards discussing sexuality or drug use with health professionals. The guide was modified and enhanced after the first 10 interviews.

Interviews were conducted confidentially in a quiet room and audio-recorded with the participant’s permission. In addition to the interviews, socio-demographic data were collected: sex, age, education level, country of origin, and reason for immigration. Date of arrival in France was collected from the second half of the sample. Most interviews (N = 30) were carried out in French, three interviews were carried out in English, and one in Arabic by a researcher who is a native speaker of this language (IBN).

### Data analysis

Interviews were transcribed verbatim and then analyzed through thematic analysis. Six researchers (SB, IBN, FT, AFD, SL, MD) worked in pairs to code a total of two interviews, and then codes were reviewed by the rest of the team to ensure consistency. This triangulation was done to ensure the validity of the results, according to a method proposed by Burnard [10]. NVivo10 Software was used (license n°12). We present the results according to the COREQ guidelines [11] in S1 Appendix.

The one interview carried out in Arabic was directly transcribed and analyzed in French by the interviewer (IBN). The three interviews conducted in English were transcribed and analyzed in English with no translation.

### Ethics statement

The STRADA study received approval from a Parisian Ethical Institutional Review Board (IRB n°00003835, protocol 2016/43NI) and was registered at the data protection agency (CNIL n°2008669). The qualitative study was conducted anonymously (i.e. no identifying data were collected). Participants who accepted to be interviewed were systematically asked if they consented to be audio-recorded at the beginning of each interview.

## Results

### Participants

Thirty four migrants were interviewed. Two migrants explicitly refused. Seven had insufficient language skills to complete the interview. The interviews lasted between 6 and 45 minutes, with an average of 15 minutes.

Of the 34 migrants interviewed, half were women (n = 17). Age ranged between 18 and 59, with an average of 32.6 years old. The participants’ region of origin was mostly Sub-Saharan Africa (n = 18; 52%). The main reason for migrating to France was family reunification (n = 17; 50%). Socio-demographic characteristics of participants are summarized in Table 1.

**Table 1:** Characteristics of participants by origin.

		Number (% total)
**Nationality**	Sub-Saharan Africa	18 (52%)
	North Africa/Middle East	6 (18%)
	Asia	4 (12%)
	Eastern Europe	3 (9%)
	South America	3 (9%)
**Reason for immigration**	Studying	6 (18%)
	Work	3 (9%)
	Refugee	4 (12%)
	Family reunification	17 (50%)
	Other	4 (12%)
**Education level**	Primary education	11 (32%)
	Completed high school	9 (26%)
	Bachelor’s degree	7 (21%)
	Master’s degree	7 (21%)
**Occupation**	Working	10 (29%)
	Seeking employment	14 (41%)
	Student or in training	9 (26%)
	Housewife/husband	1 (3%)

From the analysis, seven main themes emerged: perception of HIV/AIDS by migrants, knowledge and attitudes towards HIV screening, preferred HIV testing conditions, knowledge and perceptions of hepatitis B and C, discussing sexuality and drug use, attitudes towards rapid screening methods provided by a health professional and opinions on HIV testing during the mandatory health check-up.

### Perception of HIV/AIDS by migrants

Although all migrants knew that HIV is a sexually transmitted disease and mentioned condom use as a means of protection, a few reported beliefs such as abstinence or faithfulness as being the most important means of protection. One young woman after initially mentioning condoms as means of protection dismissed them later as unreliable as they could be pierced and highlighted faithfulness as a more reliable method.

One participant, working in a non-governmental organization in Togo, told that many people there still held erroneous beliefs about HIV transmission, such as kissing. Beyond theoretical knowledge about transmission, there were still lingering beliefs that HIV could be caused by a mystical spell.

All participants except one (a woman from Cape Verde) knew about the existence of HIV treatment. One participant only learned about the treatment after moving to France. The availability of HIV treatment in France was mentioned by several migrants, expressing trust in the French health system.

“*I think that yes*, *in France*, *it is well developed*, *everything about medicine*. *I think it can be given free of charge”(M7)*

Migrants described how antiretroviral treatment could stop the progression of the disease, thus extending life expectancy. Nevertheless, most participants viewed treatment as not very effective, burdensome, and with many side effects. One participant (a Chinese PhD student) thought that HIV treatment did not work. Because the lifelong treatment had important impacts on quality of life, they also hoped that a curative treatment would become available. Their knowledge about HIV essentially came from the media, especially television. Sometimes they received information from prevention campaigns, usually carried out in school or during cultural events (such as Carnival), from close friends or family members living with HIV.

Regardless of the level of knowledge of HIV, participants perceived HIV as a serious and eventually fatal disease. Some African migrants explained that when someone died unexpectedly, an HIV infection was often suspected due to the high prevalence of the disease. This suggests that HIV can be undiagnosed or hidden among close relatives.

*“When my husband died, everyone said he had AIDS.” (M2)*.

Participants, essentially from sub-Saharan Africa, mentioned the distrust that people in their community had towards others living with HIV. The stigma carried by people with HIV was also related to religious perceptions of marriage and sexuality, and not only in the sub-Saharan African community. For example, HIV could be explained by having forbidden premarital sex. People living with HIV find themselves rejected from society and sometimes their family–often times leading them to hide their disease.

### Knowledge and attitudes towards HIV screening

In order to optimize voluntary participation in this study, we chose to not ask direct questions related to the migrant’s own history related to HIV testing. However, the majority of participants spontaneously reported having been tested for HIV at least once in their lifetime (N = 23). Three migrants said that they had been repeatedly tested for HIV and four migrants reported that they had never been tested for HIV. The reported reasons for testing included: recent risky behavior, pregnancy, prenuptial health check-up, blood donation, surgery, or a general check-up proposed by a doctor. Two migrants mentioned receiving HIV testing as part of the migration process to emigrate to Germany or Luxemburg. For the majority of participants, HIV testing provided by physicians was carried out in systematic situations recommended by the guidelines (such as pregnancy or the legislation regarding immigration*)* without an evaluation of risky behavior.

For all participants who have been screened for HIV, the experience was positive, despite the stress of having to wait for the results (six participants).

*“About the blood sample, you can have your appointment two or three days later (for results) […] and two or three days later it’s like you are being pushed to death*. *So you get a lot of bad ideas in your head.” (M40)*

*“So the results waiting, you are stressed*, *you don’t know, you are scared, and as soon as you know the results, it gets better.” (M35)*

The benefits of being tested for HIV included: being treated if seropositive, being reassured on individual serological status, improving quality of life, and not infecting others. Several factors facilitated the decision to undertake an HIV test: not feeling personally at risk, perceiving HIV as a chronic disease like any other, having been tested positive for another asymptomatic disease (like hepatitis) and being aware of a high prevalence of the infection in their community or country of origin.

### Preferred HIV testing conditions

Some participants, who had never been tested, highlighted the necessity to be mentally prepared for the test, especially when thinking about the possibility of a positive test.

Although most participants were in favor of HIV testing, they also mentioned possible obstacles and drawbacks for other migrants to be tested. Besides the anxiety linked to the testing and the fear of a positive diagnosis, unfavorable living conditions were also mentioned. Soon after arrival in France, concerns about housing and employment took precedence while health was not seen as a priority.

*“I don’t really feel like it [doing an HIV testing] you see… Because… when someone leaves their country, they come here*, *the wait makes them focused on their [administrative] documents. So when someone will tell them ‘do you want to do an HIV test?’, they won’t even listen to you.”*

We asked the participants what the ideal conditions to undergo an HIV test were. There was a clear preference from most participants for a test conducted by a qualified and experienced health professional (doctor or nurse), especially because they would maintain medical confidentiality. Some participants had doubts about the confidentiality of charity or community organizations performing HIV rapid tests without a health professional. Some participants, mostly those with a low level of education, had a preference for a medical doctor, who were perceived as more experienced, knowledgeable and reassuring. Some migrants would prefer conducting a test with their family doctor they trust. Regarding communication, some migrants mentioned that they would like to be informed about the possibility of the screening test in advance to the medical check-up. Taking time to mentally prepare for the test was seen as essential. Most participants also expected the health professional performing the test to communicate clearly about HIV and reassure them about concerns (e.g., regarding the management of the disease, access to care, and the lack of consequences for obtaining a residence permit).

### Knowledge and perceptions of hepatitis B and C

Hepatitis was not a well-known disease among the participants, regardless of the country of origin and level of education. Even one participant living with hepatitis B had little knowledge about it. Another participant, working in a charity fighting HIV in Benin, did not know about the sexual transmission of hepatitis, despite his own involvement in HIV prevention work and his awareness that HIV prevalence was rising. Most of the participants did not know that the liver was the affected organ, or that the disease was transmittable.

*“Well*, *hepatitis…hepatitis B, other diseases are not really known to the general public. Because people have stigmatized, HIV and AIDS so much that they would rather … they see HIV and AIDS as a really awful disease. A disease…beyond that there is no other [worse] disease! But hepatitis B is also devastating! There are…there are plenty of other orally transmittable diseases that are worse, even, than AIDS.”*

Participants did not distinguish between different types of hepatitis (A, B, and C). The majority of participants described hepatitis as being transmitted by the orofecal route. Transmission by blood (e.g., sexual transmission, contaminated materials, drug use) was not mentioned.

Most of the migrants’ knowledge about hepatitis was related to vaccination, notably before travelling or before arriving in France. Among our participants, it was rare that they had knowledge of, or experience with, screening for hepatitis B or C. Screening had usually been carried out during a pregnancy or during a routine medical check-up with a physician.

All of the participants indicated that they did not have much information about these infections. They were disappointed in the lack of awareness and prevention efforts for hepatitis such as those that exist for HIV/AIDS. As such, they were interested in deepening their knowledge about hepatitis.

*“To begin with*, *HIV screening, you hear people talk about it. There are associations that everyone is aware of. But for hepatitis testing, maybe you should have more people taking talking about hepatitis testing.”*

The majority of participants perceived hepatitis as a severe illness which is possibly deadly. Acute hepatitis symptoms were mentioned most frequently: fever, jaundice, vomiting, and weakness. There were also, however, several misconceptions. In fact, whereas one migrant thought that these symptoms were related to a blood disease, another described them as a disease affecting the kidneys or the lungs. Another migrant believed that it was impossible to have hepatitis B without already having contracted hepatitis C. The chronicity of the disease was rarely mentioned: some participants perceived hepatitis C to be a more serious disease than hepatitis B. The term “hepatitis” also seemed to be associated with hepatitis A. In this context, oral transmission through contaminated water was the mode of infection most regularly mentioned.

Despite the limited knowledge about hepatitis, there were no particular worries about the availability of a treatment. The possibility that a treatment existed and the fact that one could benefit from such treatment in the case of hepatitis, participants did not seem concerned about the availability of treatment. Although it was considered as a serious disease, hepatitis was not considered as a “plague” like HIV. Most participants, considered hepatitis as an ordinary disease that required treatment. Only two participants mentioned the idea of lifelong treatment. One Russian participant, had a high level of awareness in terms of sexually transmitted diseases; the other participant had worked in a hospital.

### Discussing sexuality and drug use

Few participants mentioned having discussed their sex life or sexual activity with a health professional. In contrast to men, women were more spontaneous and more at ease when raising the subject of sexuality with their healthcare professionals. In our sample, only women had taken the initiative to ask questions about sexual matters to their doctor, sometimes during a gynecology consultation. One woman wished to receive information on sexually transmitted diseases. Another had approached a health professional to discuss sexual pleasure and her genital mutilation. Only few male participants mentioned being asked about their sexuality during a medical visit in the past. The majority of participants expressed disappointment that their doctor had never addressed the subject of sexuality during a consultation. Other participants saw no barriers to having a doctor discussing sexual matters with them.

*“Yes*, *for me I say it’s good*, *because there are many women or men with sexual problems but they don’t know how to explain them or talk about them*. *So you know it’s a sexual problem*, *but it’s not easy to bring it up directly*. *But if there were someone who was used to talking about them…so even when you’re talking with people and all of a sudden you notice that this person or that person*, *you could talk with them*. *But if you go to the doctor’s office and if the doctor doesn’t ask (laughs)*… *But for me*, *since I arrived here*, *nobody has talked to me about these things*.*”* (M2)

*“It’s a benefit*. *Because knowledge is power, as they say. Knowledge is power. The fact of knowing certain things, like maybe one heard but didn’t pay attention. Such as when you come to a specialist with expertise, who can better explain [health] problems, and the pros and cons, finally, when you leave there, you leave with knowledge. And tomorrow, it can…it can help you. It can help you, learning something, it can help you.”*

Even though the majority of migrants that we interviewed had a favorable response towards discussing sexuality at OFII, some limitations were noted. Embarrassment talking about sex, especially with an unfamiliar healthcare professional, was mentioned. Two participants (one man, one woman) preferred to have these questions discussed with their trusted family doctor.

Only one participant indicated that it was not necessary for healthcare providers to address sexual questions, whether or not they were from OFII. He felt that this was a private matter and that these matters should only be discussed with specialists (e.g., sex therapists).

The obstacle to discussing sexuality at OFII could be a lack of sincerity and/or openness among migrants about their sexuality due to fear of not obtaining a residence permit.

*"In truth I think that it’s to say*: *what will happen…to the person who says that? For example if I come here and you ask me “Do you have sexual relations?”, that is not going to interfere with my visa, but if you have [certain] sexual relations like that, if it’s positive, we have a treatment for you. If you use drugs and you want to get off them, if it bothers you, if it makes you sick, or something like that, we have people who will help you.”*

The acceptability of discussing drugs use at OFII was high. Even if participants were not personally concerned, they did not feel uncomfortable with the idea of talking about this issue. Thus, they did not have problem talking about it with their healthcare provider during a medical visit in the past. They indicated that they would gladly accept information on the subject. Addressing drug use was considered an integral part of the doctor’s role. Talking about drug use could allow the doctor to gain a better global understanding of the patient. However, a majority of participants indicated that their doctor had never discussed drug use with them. According to them, in order for this experience to be considered beneficial, and well perceived by migrants, it must be associated with prevention that would lead to adequate treatment. This approach should also target young people, considered to be the principal victims of drug abuse.

Nevertheless, despite good individual acceptance of discussing drug use in our sample, the participants recognized suspicion about the sincerity and openness of other migrants when discussing drug usage at OFII, expressing their eventual fear of the negative consequences for not obtaining a residence permit.

*“No, it doesn’t shock me, because the doctor asks me about it for my health. For example, if you don’t sleep well, it can happen that you take drugs and arrive in the hospital. If the doctor wants to know what really happened, they’re going to ask you some questions. It can’t shock you*, *it’s normal for them, it’s part of their work.”*

*“If it’s really important*, *you need to especially talk to young people, the youngest I think, especially to explain to them that it’s not a taboo here, they really have possibilities, if they are addicted and there are real solutions that they can know about, they might not have them in their country.”*

### Attitudes towards rapid screening methods provided by a health professional

Amongst the 34 participants, 11 had never heard about a rapid HIV test and only a few knew how it worked. Some participants understood them as the self-test kits are sold in pharmacies. The most aware of HIV rapid tests were migrants from Africa who were less than 35 years old. Some of them said that rapid tests were more widespread in their home country than in France. Men concerned with HIV prevention were more interested in using them. When we explained briefly those tests, all participants wanted to learn more.

The benefits for using rapid tests mentioned by participants were: the immediacy of results, reduced stress while waiting for results, the simplicity, as it could replace blood drawing, and the convenience that there was no need to obtain results, thus saving time and transportation costs.

Rapid tests were also perceived as having disadvantages. Firstly, the reliability of those tests was seen as weaker due to the rapidity of results and because of their perceived novelty. Classic blood tests was seen as the “gold standard” and often preferred. Even after explanation of the reliability of rapid testing, some doubts persisted. Secondly, some migrants preferred the classical test, as several diseases could be tested with the same sample. Finally, two African women mentioned having a problem with the rapidity of results. Receiving a positive result right after performing the test was described as brutal and a source of anxiety. Getting prepared for a positive result required time, especially if the tested person is at risk of being seropositive.

*“As for me, if I don’t feel confident or if I have doubts*, *knowing me I would rather do the blood draw because I would be scared to be told straight away. I would rather wait […]. If you do it for a birth, for example, you can tell me straight away but if I have doubts I prefer the blood draw”*

*“If you want to have the results straight after*, *if you’re confident that the test will be negative, you can have the results straight away, you will be satisfied. But if you are not sure of yourself, and that you want the results straight away, and that it is positive, you will be unhappy […]. You need to prepare mentally and physically to accept the results of this test.”*

In conclusion, rapid tests were perceived as mostly beneficial because of the immediate results, but they were sometimes described as fear inducing. Thus, they should be administered along with opportunities for counselling, reassurance, and follow up.

### Opinions on HIV testing during the mandatory health check-up

Many participants said they would accept rapid HIV and hepatitis testing during the check-up if it was offered. Five participants would have not accepted.

Some migrants said that testing at OFII was “convenient” because they could get tested without having to go to another screening center, saving them time and transportation costs. It was particularly convenient because many migrants did not necessarily know where to get tested. The medical check-up was seen as an entry point into the French health system. Some participants felt that France as a state had the right to know the HIV status of migrants, therefore testing at OFII was justified. One man thought that testing should be compulsory and performed before migration.

Among the 34 participants, five of them did not wish to be tested for HIV or hepatitis at OFII. One reason was the lack of familiarity and trust in the health professionals at OFII. For one woman, the ideal setting for HIV testing was with the family doctor. Other migrants felt uneasy discussing intimate matters related to sexuality with unknown health professionals. A man from sub-Saharan Africa, involved in HIV prevention NGO campaigns in his country, was opposed to HIV screening, fearing that other migrants could feel obliged to do the test. For some participants, the medical check-up was a bad time for an HIV test. Deciding to do a test for infectious or other diseases required time to think before. Participants mentioned that newly arrived migrants did not have the time and availability to think it over, because of other priorities such as administration, housing, or finances. They indicated that a better time for these tests was after resolving those issues.

In conclusion, participants felt free to refuse the screening. The barriers were more often expressed for others than for themselves, especially if being a member of NGO. Not accepting HIV screening at OFII seemed to be related to either the discomfort of discussing sexual issues with an unknown health professional or the anxiety of a positive diagnosis especially in the context of migration (e.g., concerns about the residence permit, other administrative concerns). These anxieties could be related to the lack of knowledge about the availability of HIV treatment. Also, especially for migrants from sub-Saharan African, stigmatization around HIV was present.

We asked migrants how they felt about the medical check-up at OFII besides testing for HIV or hepatitis. The medical check was appreciated, as it reflected the concern of the French government for migrants and the opportunity to get informed about the French health system. However the medical check-up was also described as incomplete and sometimes too fast. One participant wished that the medical analyses performed at OFII could be extended to more diseases. The medical check-up was described as stressful by some participants as the delivery of residence permit was dependent on its completion. For other migrants, it was described as a simple procedure.

The participants suggested ways to increase the acceptability of rapid tests. They suggested highlighting the voluntary nature of the tests, and that a positive test result does not have an impact on receiving the residence permit. One participant suggested that informing migrants before the medical check-up could help migrants be prepared for it.

## Discussion

### Main results

The objective of this qualitative study was to explore the perceptions on testing and rapid testing and opinions on offering testing at OFII during the medical check-up amongst legal migrants undergoing the medical check-up. We have found a high level of acceptability amongst participants. The mentioned reasons for accepting the tests were a benefit for their health, avoiding transmission, and access to care. The reported barriers to testing were: anxiety, fear of a positive result, not feeling at risk, and not feeling prepared for a test. The specific barriers to HIV and hepatitis testing at OFII were the uncertainty about its compulsory nature and about possible consequences on the residence permit. When educated about those issues, the acceptability of testing was high. Some migrants thought that is was better to have time to prepare mentally when the practical issues related to the immigration were resolved. However to find a stable social and economic situation can take years and by this time the disease can progress.

Migrants were relatively well informed about HIV, despite poorer knowledge about the efficacy of treatment, but much less informed about hepatitis. Rapid tests were not very well known. After having been informed about the details of these tests, participants expressed an interest in using them due to the rapidity of the results. However, some participants preferred classical blood tests, perceiving them as more reliable, and also preferring to avoid getting the results immediately due to feelings of discomfort about receiving a positive result.

Finally, feeling compelled to do the test is not specific to this setting which remains anonymous. Indeed, HIV testing is offered in a variety of situations, including in migrants’ communal housing [12]. In those close-knit settings, migrants might not feel free to refuse testing.

### Comparison with literature

A study conducted in the Netherlands found an acceptability rate of 54% for HIV testing [13]. In our study, 29 out of the 34 participants included said they would accept a HIV test performed at OFII. Our study sample is too small to allow generalizability, so more work is needed on the acceptance of hepatitis and HIV tests among migrants.

Other studies in migrants have also suggested a high level of acceptability for screening for infectious diseases [14].

Studies have found that lack of access to treatment is an important barrier for HIV testing for migrants in high-income countries [15]. In France, everyone living with HIV can access free treatment, regardless of income or migration status. Thus, access to treatment is not a barrier to testing. However if migrants are not aware of this access to treatment, the barrier remains. In our study, most migrants knew about the existence of treatment and its free access in France. However, there were areas where awareness could be improved for migrants. A study published in 2007 amongst sub-Saharan African migrants living in the greater Paris area found that even educated migrants were significantly less informed about the existence of treatment than French citizens [16,17]. Therapeutic care for HIV has been improved, with reduced side effects and fewer pills needed per day [18]. Educating migrants about treatment will be important for guiding them to appropriate screening, prevention, and treatment.

We found a low level of knowledge about hepatitis amongst participants. A narrative systematic review of studies conducted amongst migrants also found a low level of knowledge of hepatitis, including confusion between hepatitis A, B, and C, or the belief that A, B, and C were different stages of the same disease [19]. Similar results were found in the general population in France in 2010 [20]. Another study found confusion between hepatitis and jaundice [21], and in another study Turkish migrants in the Netherlands did not know that hepatitis B could be transmitted sexually, which might explain the low level of stigmatization of this disease [22]. The lack of knowledge on hepatitis is widespread. Given the burden of hepatitis on population health, more information, education, vaccination, and screening test campaigns are needed.

We found that participants felt that the medical check-up at OFII was incomplete. Migrants in Sweden were similarly disappointed that the screening focused on infectious diseases and overlooked mental health, or that the screening was performed only once [23]. Another study in Sweden found that migrants had a positive perception of the health examination and trusted health professionals, but found that it was too restrictive, especially in terms of dental care or mental health [11]. Recently mental health screening was added to the OFII medical visit.

In the STRADA study we found a high level of acceptability and interest in rapid tests. Rapid tests can benefit those who do not have easy access to the health care system. Receiving an immediate result reduces the number of people who never come back for their results. Rapid tests are increasingly being used in France, mostly by communitarian organizations that perform peer HIV screening. In France in 2014, 28% of all people receiving a rapid test for HIV were migrants [24]. A study conducted amongst the general population consulting their family doctors in France also found that rapid tests were readily accepted [25].

Some participants from the sample have been tested for HIV and hepatitis per their doctor’s suggestion in situations where screening is recommended, such as pregnancy. Besides those situations, testing was rarely proposed by doctors who scarcely evaluated risk factors of transmission. In the majority of cases, screening was initiated by the migrants. A study of the missed opportunities for testing found a poor evaluation of transmission risks by doctors and a fear of stigmatizing migrants when offering HIV screening [26]. Another qualitative study, conducted by our team amongst the health care staff of the OFII centers also reported a fear of stigmatizing migrants as a barrier to the implementation of testing [27]. A study conducted amongst general practitioners also found this fear of stigmatization, as well as a risk of deteriorating the physician-patient relationship as barriers [28]. The results of our study show that this fear is unfounded and migrants do not feel stigmatized by merely offering such a test.

In recent years there has been a shift in the paradigm for HIV testing, moving from patient-initiated testing to provider-initiated testing [29]. This change in paradigm ensures that people who would not have come forward for a test due to barriers including a lack of information, not thinking of themselves at risk, or feeling too embarrassed to ask for a test. Provider-initiated testing is particularly appropriate for migrants, especially because one of the reasons for late diagnosis is people not thinking of themselves as at risk [30]. Indeed, some studies conducted amongst migrants suggested that the provider inviting for a test facilitated the acceptance of testing [21], especially when some migrants felt uncomfortable about explaining the reasons why they wanted a test [31] or when taking the initiative of getting tested might be viewed with suspicion by other people in the community [22]. Our results suggest that it is crucial to encourage more health care providers to initiate the offer of testing, therefore validating the rationale for the STRADA project.

The STRADA study includes a questionnaire of risk factors including questions related to sexuality and drug use. Thus, we explored the acceptability of discussing these subjects with doctors. Participants had no difficulties talking about drugs, but it may be that some migrants outside of our study would have reticence about discussing drug use. Regarding sexuality, participants stated that this was a part of routine medical counseling. Young men seemed less comfortable discussing sexuality. Women seemed to have more opportunities to discuss sexual health with their family doctor, through contraception or pregnancy. Other studies have found similar results. A Swiss study found that 85% of participants would not be embarrassed if their physician would ask sex-related questions, and of the 15% that would be embarrassed, 76% would nevertheless prefer their physician to ask those questions. This highlights the gap between patients’ wishes and doctor’s practice of discussing sexuality [32]. Another study found 88% acceptability of a risk factor questionnaire for HIV, HBV, and HCV in a family practice. The main barrier to discussing sexuality was related to doctors [33].

### Strengths and limitations

Our study is the first to explore the acceptability of testing at OFII centers in France, as existing qualitative or quantitative studies have explored general barriers to testing for HIV and hepatitis for migrants. The main strength of our study is that we included a diverse range of participants. The diversity in terms of age, origin, gender, and educational level ensured a diversity of perspectives, supporting the external validity of results [34].

To improve the quality of the study the team made several revisions to the interview guide and the interviews were coded in pairs. Then the rest of the team reviewed the codes and suggested modifications. This triangulation ensures that the subjectivity in coding is limited and improves the validity of the findings [10].

It is possible that some biases are present based on our method of recruitment. The study was done both on an “opt-in” basis (migrants interested in the study had to come forward) and on an “opt-out” basis (after the medical check-up, migrants were all invited to participate unless they were not interested), depending on the logistics of the OFII center. The opt-in format might have recruited more participants knowledgeable about HIV and hepatitis. Perhaps these participants were more comfortable talking about sexuality and infectious disease, and possibly more interested in testing for those diseases than other migrants. However on many visits, an opt-out format helped us get a more representative view of the migrants undergoing the medical check-up. It is possible that practicalities like longer visit times or wait times would reduce the acceptability of rapid testing.

About half of participants were from sub-Saharan Africa, although they made up only 16% of all migrants who underwent the medical check-up at the OFII in France in 2017 [35]. This might be because they more often speak French, relative to other migrants, or because they are more comfortable speaking about HIV. A study evaluating voluntary HIV testing amongst migrants in the Netherlands found that migrants who came from a country with a high HIV prevalence were more likely to accept testing [13]. We conducted the interviews in French, English and, for one interview, Arabic. Thus, our study is not representative of all migrants. Although we could have used interpreters, this method could have created another bias: participants might have changed their answers or omitted certain topics. Of the four interviews carried out in languages other than French, one (in Arabic) was translated directly to French by the interviewer who is a native speaker and the three interviews carried out in English were not translated and were analyzed as such by the researcher who have a proficient level of written English. Despite the proficiency of the research team in either of those languages, it is possible that some level of bias or misinterpretation remain. However, carrying out interviews in different languages allowed us to increase the diversity of migrants included, and therefore the validity of results, and to include migrants who might be less likely to ask for or be offered a screening test for HIV, HBV, and HCV. Indeed, studies suggest that migrants’ lack of proficiency in the language of the country of origin is a barrier to screening for HIV, HBV, and HCV [36–41]. Therefore, it is likely that regarding results validity, the advantages of carrying out interviews in several languages have outweighed the risks of bias. This language barrier related to screening will be further explored in future studies carried out by the research team.

Our research team received some feedback that migrants cannot freely express their opinion about acceptability and that they feel forced to accept the screening. Our study sought to gather important information directly on this issue by talking with migrants directly. Indeed, despite being subjective, patients are the best experts to assess their health and impact of a treatment on their symptoms or health-related quality of life (compared to the own subjectivity of doctors or experts) Similarly, Patient-Reported Outcomes (PROs) are now well recognized endpoints by medical regulatory agencies [42,43].

### Policy implications

Our study shows that HIV and hepatitis tests at OFII are acceptable to migrants, provided that certain conditions are met. The most important points are that migrants are fully aware that the test is voluntary and that the results of screening are unrelated to the residence permit. Providing clear and comprehensive information to migrants is crucial. By recognizing that health literacy can be limited among migrants, better methods and precautions can be devised to maximize the efficacy and equality of the healthcare system [44].

We found that some of the barriers to testing at OFII (such as the fear of stigmatization or difficulties in discussing sexuality) are more related to health care providers, rather than aspects of the migrant population. Thus, it is crucial, when that health care providers are aware of barriers related to rapid tests so that they can effectively counsel their patients.

## Conclusion

The objective of this qualitative study was to explore the acceptability and feasibility of performing voluntary testing for HIV, hepatitis B, and hepatitis C at OFII centers during the medical check-up. We have found a high level of acceptability despite some barriers that can be addressed. Although tests for infectious diseases are currently offered at OFII during the STRADA study, their acceptability should be evaluated quantitatively in future studies. Despite experts’ recommendations, migrants are not routinely screened for HIV in France. Health authorities should take advantage of results from the STRADA study to propose systematic screening procedures. It would be a wise public health decision for the good of this population at risk for infectious disease.

## Supporting information

S1 Appendix COREQ guidelines. (PDF)
