# Supplementary material for: Good acceptability of HIV, HBV, and HCV screening during immigration medical check-up amongst migrants in France in the STRADA study
Source: PLoS One. 2020 Jun 26;15(6):e0235260. doi: 10.1371/journal.pone.0235260 (PMC7319329; doi:10.1371/journal.pone.0235260)
Supplement: S1 Appendix — (PDF) [file pone.0235260.s001.pdf]

Additional file 1: COREQ Guideline checklist

**Domain 1 : Research team and reflexivity**	**Personal characteristics**	
	1. Which author(s) conducted the interview?	The interviews were conducted by FT, AFD, SB, IBN, and SL.
	2. What were the researcher's credentials?	MD: Medical Doctor and PhD; FT: PhD in public health, AFD: Medical Doctor and PhD candidate, SB and IBN: Medical doctors, SL: Masters in clinical research, LCB: Medical Doctor and, PhD, FL: Medical Doctor and PhD, SD: Medical Doctor and PhD, TLL: Medical doctor, OC: Medical Doctor and PhD
	3. What was their occupation at the time of the study?	OC, MD, FT, AFD, SL were employed at the research unit; IBN and SB were residents carrying out an internship at the research unit. All other researchers are associate researchers to the research unit.
	4. Was the researcher male or female?	MD and OC are male, all other authors are female.
	5. What experience or training did the researcher have?	All researchers have been trained in qualitative research prior to the start of the study.
	**Relationship with participants**	
	6. Was a relationship established prior to study commencement	No relationship was established before starting the study.
	7. What did the participants know about the researcher?	Participants didn't know anything about the researchers.
	8. What characteristics were reported about the interviewer/facilitator?	No characteristics were reported. Participants only knew that the researchers didn't work for OFII.
**Domain 2: Study design**	**Theoretical framework**	
	9. What methodological orientation was stated to underpin the study?	Phenomenological study using grounded theory analysis.
	**Participant selection**	
	10. How were the participants selected?	All adult participants who agreed to participate and understood the study goals were selected.
	11. How were the participants approached?	Two different approaches were used, depending on the OFII centre in which the study was carried out. The two approaches

		are described in the methodology section.
	12. How many participants were in the study?	There are 34 participants in the study.
	13. How many participants refused to participate or dropped out? Why?	Two participants explicitly refused to take part in interviews. Reasons are detailed in the results section of the article.
	**Setting**	
	14. Where was the data collected?	The interviews were carried out in a private space in an OFII centre.
	15. Was anyone else present besides the participants and researcher?	Some interviews were conducted with two researchers.
	16. What are the important characteristics of the sample?	Participants are a diverse sample of migrants undertaking the medical check-up at OFII
	**Data collection**	
	17. Were questions, prompts, guides provided by the author? Was it pilot tested?	The questions were asked following an interview guide. The interview guide was prepared in the whole team. It was pilot tested then adjusted.
	18. Were repeat interviews carried out? Details	No repeat interviews were carried out.
	19. Did the researcher use audio or visual recording to collect the data?	All interviews were audio recorded with the consent of the participants.
	20. Were field notes made during and/or after the interview or focus group?	Notes were taken during interviews.
	21. What was the duration of interviews or focus groups?	The interviews lasted between 6 and 45 minutes, with an average of 15 minutes.
	22. Was data saturation discussed?	Data saturation was discussed with the entire team of researchers.
	23. Were transcripts returned to participants for comments and/or correction?	No transcripts were returned to participants.
**Domain 3: Analysis and findings**	**Data analysis**	
	24. How many data coders coded the data?	The original coding tree was created with the entire team of researchers. Then, two researchers (IBN and SB) independently coded the transcripts.

	25. Did authors provide a description of the coding tree?	The coding tree is not described.
	26. Were themes identified in advance or derived from the data?	Themes were derived from data.
	27. What software, if applicable, was used to manage the data?	Researchers coded using the NVivo 10 software.
	28. Did participants provide feedback on the findings?	The participants didn't provide feedback on the findings.
	**Reporting**	
	29. Were participant quotations presented to illustrate the themes/findings? Was each quotation identified?	Some participants quote are added to illustrate findings.
	30. Was there consistency between the data presented and the findings?	The data presented and the findings are consistent.
	31. Were major themes clearly presented in the findings?	The major themes are presented in the findings.
	32. Is there a description of diverse cases or discussion of minor themes?	Minor themes are discussed.
